# Emerging Therapeutic Strategies in Oral Cancer: Epigenetic, Mitochondrial and Immunotherapy Approaches

**DOI:** 10.1111/jcmm.71107

**Published:** 2026-04-24

**Authors:** Geetha Shanmugam, Carmelin Durai Singh, Rekha Thiruvengadam, Muthu Thiruvengadam, Vitaly Morozov, Roman Kolesnikov, Ali Alkaladi, Rashad Saleh, Mohammad Ali Shariati

**Affiliations:** ^1^ Center for Global Health Research, Saveetha Medical College and Hospital Saveetha Institute of Medical and Technical Sciences Thandalam, Chennai Tamil Nadu India; ^2^ Microbiology and Nanoscience Lab‐Helix Research Studio, Department of Neonatology, Saveetha Medical College and Hospital Saveetha Institute of Medical and Technical Sciences Thandalam, Chennai Tamil Nadu India; ^3^ Department of Applied Bioscience, College of Life and Environmental Science Konkuk University Seoul Republic of Korea; ^4^ Centre for Research Impact & Outcome Chitkara University Rajpura Punjab India; ^5^ Saints Petersburg State Agrarian University Saints Petersburg Russia; ^6^ Department of Biological Sciences, College of Science University of Jeddah Jeddah Kingdom of Saudi Arabia; ^7^ Medical Microbiology Department, College of Sciences Ibb University Ibb Governorate Yemen; ^8^ Kazakh Research Institute of Processing and Food Industry Semey Branch of the Institute Almaty Kazakhstan

**Keywords:** epigenetic alterations, mitochondrial dysregulation, nanoparticle‐based drug delivery, oral cancer, RNA interference, tumour microenvironment

## Abstract

Oral squamous cell carcinoma (OSCC) is the most common type of oral cancer and poses treatment challenges owing to genetic, epigenetic and environmental factors. Conventional treatments, including surgery, chemotherapy and radiation therapy, often have limitations in terms of efficacy and tolerability. Advances in epigenetic therapies such as DNA methyltransferase and histone deacetylase inhibitors offer promising avenues for reversing abnormal gene expression in OSCC. Mitochondria‐targeted therapies leverage metabolic disruption and reactive oxygen species modulation to induce apoptosis. Immunotherapy, particularly with immune checkpoint inhibitors and cancer vaccines, enhances the immune response against cancer cells. This review explores the interplay between the tumour microenvironment and oral microbiome in OSCC progression and treatment response. Additionally, RNA interference therapy and nanoparticle‐based drug delivery systems enable targeted therapeutic strategies, reduce off‐target effects and improve efficacy. Although these approaches show potential, challenges in clinical translation remain. The integration of precision medicine with innovative drug delivery systems can significantly improve patient outcomes in oral cancer management.

## 1 Introduction

Over the past several decades, the scope of cancer treatment options available to patients has been narrow. These options primarily include surgery and radiation therapy for solid tumours confined to specific areas, along with chemotherapy for blood‐related cancers and metastatic solid tumours [1]. Solid tumours, such as OSCC and breast and lung cancers, require multimodal treatments including surgery, radiation and chemotherapy, often targeting tumour microenvironments and angiogenesis. In contrast, non‐solid tumours (e.g., leukaemia and lymphoma) rely on systemic therapies such as chemotherapy and immunotherapy because of their diffuse nature, making localised treatments less effective. However, the emergence of targeted therapies has shifted the focus to understanding the biological mechanisms underlying the response to and resistance to these agents. Nevertheless, while conventional therapies such as targeted therapies, radiation therapy and chemotherapy primarily target epithelial cancer cells, cancer progression is not solely driven by changes within cancer cells. It also involves interactions with the tumour microenvironment (TME), as well as alterations in cellular metabolism and immune response [2]. The TME is a dynamic network of cellular and molecular components, including fibroblasts, immune cells, extracellular matrix and signalling molecules that influence tumour growth, metastasis, and therapeutic resistance [3]. The TME plays a crucial role in shaping tumour behaviour and modulating treatment responses, making it a promising target for novel therapeutic interventions [4]. Oral cancer encompasses tumours that arise in various regions of the oral cavity, including the lips, hard palate, tongue and buccal mucosa [5]. The predominant form, OSCC, accounts for over 90% of cases and exhibits squamous differentiation from mucosal epithelium. OSCC is the sixth most prevalent cancer globally, with a 5‐year survival rate of approximately 50% [6]. In 2018 alone, 354,864 new cases of lip and oral cavity cancer were reported, resulting in 177,384 deaths [7]. Table 1 represents OSCC mortality by region, whereas Table 2 presents a comparative analysis of OSCC treatment modalities with their efficacy, survival benefits and toxicity profiles.

**TABLE 1:** OSCC mortality rates by region.

Region	Annual incidence (per 100,000)	5‐year survival rate (%)
North America	4–6	55–65
Europe	5–7	50–60
South Asia	8–12	40–50
Africa	3–5	35–45

**TABLE 2:** Comparative analysis of OSCC treatment modalities: Efficacy, survival benefits, and toxicity profiles.

Therapy type	Response rate (%)	Survival benefit	Toxicity
Chemotherapy (e.g., Cisplatin, 5‐FU)	30–40	Moderate, but high recurrence	High (nausea, myelosuppression)
Immune checkpoint inhibitors (Nivolumab, Pembrolizumab)	40–50	Significant improvement in overall survival	Immune‐related adverse events (colitis, rash)
Cancer vaccines (HPV‐targeted, peptide‐based vaccines)	20–30	Long‐term immune memory potential	Low, but slow onset of response

OSCC aetiology involves a combination of genetic, epigenetic and environmental factors [8]. Excessive alcohol consumption and tobacco use are major contributors to OSCC, alongside human papillomavirus (HPV) infection and disruptions in circadian rhythms [9]. OSCC presents with a range of clinical manifestations, including non‐healing ulcers with persistent pain or discomfort as well as precancerous lesions such as leukoplakia and erythroplakia, which have the potential to progress to malignancy. Patients may also develop induration and nodular growth within the oral cavity [10]. In advanced cases, OSCC can cause dysphagia and speech impairment, significantly affecting quality of life. In addition, regional lymphadenopathy is often observed, indicating possible metastasis and disease progression [11]. OSCC can present in multiple forms, including polypoid, fungating, ulcerative and infiltrative. These variations influence the treatment strategies, with more aggressive infiltrative tumours requiring multimodal approaches [12]. While conventional therapies, such as surgery, chemotherapy and radiation therapy, have made significant strides in treating oral cancer, they are accompanied by notable drawbacks and adverse effects [13]. Additionally, challenges such as low solubility, poor permeability and limited bioavailability further hinder the efficacy of oral chemotherapy [14]. Hence, there is an urgent need to develop novel therapeutic strategies or modifications to existing approaches to improve outcomes and survival rates in patients with oral cancer.

This review comprehensively analyses emerging OSCC therapies, including epigenetic, mitochondrial‐targeted and immunotherapeutic approaches. By exploring their mechanisms and translational potential, this study highlights the key advancements, challenges and future directions for personalised treatment. Unlike previous reviews that focused on single modalities, this study integrated these therapeutic strategies, providing a holistic perspective on their interplay in cancer progression and resistance. Additionally, it critically examined translational barriers and clinical applications, offering a roadmap for advancing OSCC treatment.

### 1.1 Epigenetic Based Therapy for Oral Cancer

Epigenetic alterations that affect tumour suppressor genes and oncogenes play a significant role in the progression of oral cancer. Methylation of certain genes contributes to malignant transformation, while deacetylation of both histone and non‐histone targets further facilitates oral cancer progression. Analysis of the methylation patterns of these genes can aid in the early detection and diagnosis of OSCC, representing a crucial aspect of modern research [15]. The exploration of epigenetic modifiers, including DNA methyltransferase (DNMT) inhibitors and histone deacetylase (HDAC) inhibitors, has opened up promising therapeutic avenues for OSCC [16]. These drugs have shown the potential to reverse abnormal epigenetic patterns observed in OSCC cells, leading to reactivation of tumour suppressor genes or silencing of oncogenes [16]. Oncogene silencing in OSCC primarily occurs through DNA methylation (inhibiting transcription factor binding), histone deacetylation (condensing chromatin structure) and RNA interference (RNAi) mechanisms, leading to targeted gene suppression [17]. DNMT inhibitors, such as Decitabine and HDAC inhibitors, such as vorinostat, exemplify epigenetic drugs that restore tumour suppressor activity by reversing these alterations [18].

The combination of DNMT inhibitors with conventional chemotherapeutic agents has been investigated to enhance the treatment efficacy. For example, Zebularine, a DNMT inhibitor, has been shown to significantly increase the apoptotic activity induced by cisplatin treatment, potentially augmenting the effectiveness of chemotherapy in inducing cancer cell death [19]. Similarly, 5‐aza‐CdR has been shown to reverse methylation and restore the expression of p16INK4a in OSCC cells [20]. HDAC inhibitors such as Trichostatin A (TSA) selectively target specific HDACs in OSCC, inhibit cell growth, induce cell cycle arrest, and trigger apoptosis by influencing the expression of key proteins, such as p21WAF1, Cyclins, Bax and Bcl‐2 family members [21]. Butyric acid derivatives, such as phenylbutyrate, exhibit beneficial effects by promoting DNA repair and cell survival, reducing oxidative stress and lowering the risk of OSCC and tumour progression when used alongside radiotherapy [16]. Sodium butyrate has also been shown to inhibit OSCC cell proliferation and induce cell cycle arrest [22]. Recent clinical trials have evaluated the efficacy of epigenetic modulators in OSCC treatment. For example, a phase II clinical trial (NCT03013676) investigated the use of Decitabine, a DNA methyltransferase inhibitor, in combination with chemotherapy and showed improved tumour response rates [23]. Similarly, Vorinostat, a histone deacetylase inhibitor, has demonstrated the potential to reverse chemoresistance in patients with OSCC (NCT00731736) [24]. These studies highlight the translational potential of epigenetic therapies in the clinical setting. Table 3 now includes a summary of key findings, highlighting each epidemic's mechanism of action and its potential clinical implications.

**TABLE 3:** List of epidrugs in cancer treatment.

Group	Epidrugs	Target	References
HDAC inhibitors	Romidepsin, Vorinstat, Trichostatin A, Entinostat, Panobinostat	Class I, II, IV HDACs	[25]
DNMT inhibitor	5‐azacytidine, 5‐aza‐2′‐deoxycytidine	DNMT1	[26]
HAT inhibitors	Anacardic acid, curcumin, Garcinol	p300, CBP, PCAF	[27]
KMT inhibitor	3‐Deazaneplanocin A, Pinometostat, GSK126	EZH2	[28]
KDM inhibitor	ORY‐1001, GSK2879552, GSK‐J1	LSD1, JMJD3	[29]

The intricate regulation of epigenetic mechanisms, including DNA methylation, histone modifications, and specific microRNAs, is closely intertwined with both the early and advanced stages of oral cancer. This correlation underscores the potential of epidrug therapy as an interventional strategy. The presence of discernible epigenetic markers in oral lesions, cancers and tumour‐associated mucosa underscores their significance as biomarkers, and highlights the therapeutic potential of epidrugs in enhancing patient management. Understanding the role of epigenetic alterations in diagnosis and therapeutics, particularly in cancers associated with chemoresistance, is crucial. These epigenetic modifications hold promise for the diagnosis, treatment and prognosis of OSCC patients. Consequently, an increasing focus is being placed on the development of epigenetic medicines, including novel drugs such as epidrugs targeting identified epigenetic biomarkers, which could serve as an adjuvant therapy to chemotherapy in OSCC.

### 1.2 Mitochondrial Based Therapy

The significance of mitochondrial genes in cellular energy production, apoptosis and reactive oxygen species (ROS) generation cannot be overstated. Dysregulation of these genes has been implicated in various diseases including cancer. Studies have highlighted mitochondrial gene dysregulation in oral cancer and underscored its potential involvement in disease progression [30]. Recently, there has been growing interest in exploring the connection between mitochondria and therapy for OSCC. Tumours with aberrant metabolism have highlighted the mitochondria as a key target. Mitochondria play a significant role in tumorigenesis through various pathways such as ROS accumulation and regulation of cancer stem cells (CSCs) [31]. They also contribute to metastasis by influencing the motility, invasion, and TME. Consequently, targeting mitochondria has become a potential therapeutic strategy [32].

**FIGURE 1:**
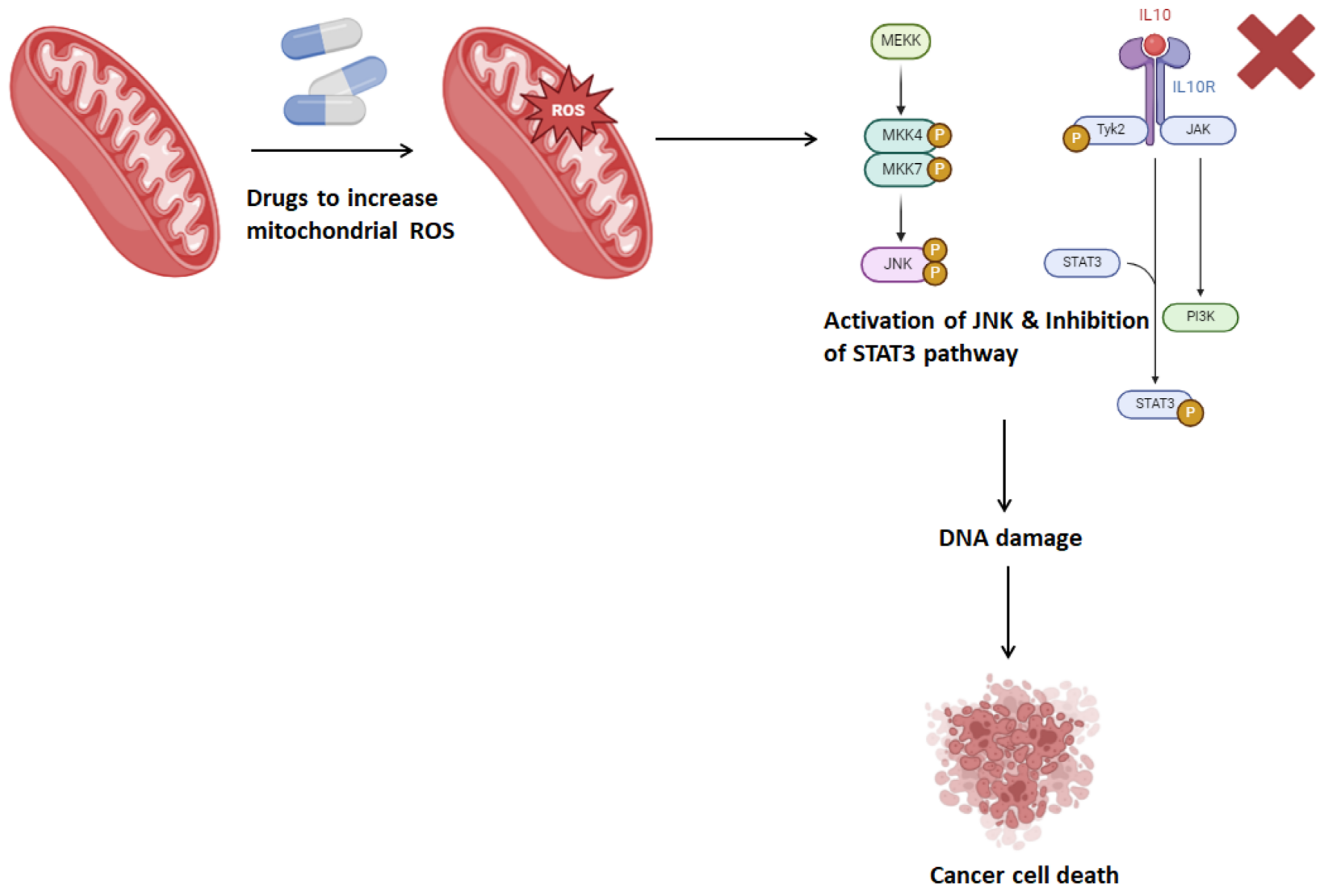
Mitochondrial‐targeted therapeutic strategies for OSCC. This strategy involves administering drugs that increase the production of mitochondrial ROS within tumour cells. This heightened ROS production induces DNA damage, ultimately resulting in cancer cell death. This figure illustrates the key mitochondrial pathways targeted in OSCC treatment. Mitochondrial apoptosis pathway (MAP)—Activation of Cyt C release triggers caspase‐mediated apoptosis. ROS modulation—Increased mitochondrial ROS can induce DNA damage and promote cell death, while antioxidant defences contribute to chemoresistance and metabolic reprogramming—Altered oxidative phosphorylation (OXPHOS) and glycolysis influence tumour proliferation and drug resistance.

For instance, the downregulation of mitochondrial calcium uniporter expression impairs OSCC cell proliferation and migration [33]. Furthermore, the oxidation of nicotinamide adenine dinucleotide (NADH) during mitochondrial oxidative phosphorylation (OXPHOS) promotes cancer cell proliferation [34]. Additionally, the M2 isoform of pyruvate kinase (PKM2), a regulator of mitochondrial function, correlates strongly with OSCC tumour progression [35]. Regulation of the mitochondrial apoptosis pathway (MAP), ROS levels and mitochondrial fission are key strategies for supporting OSCC treatment [36]. Moreover, mitochondrial ROS (mtROS) can exert therapeutic effects by interacting with various molecules and activating signalling pathways, such as c‐Jun N‐terminal kinase (JNK) and adenosine 5′‐monophosphate (AMP)‐activated protein kinase (AMPK), leading to apoptosis and growth inhibition in OSCC cells [37]. Additionally, mtROS‐induced DNA damage contributes to cell death, including in OSCC CSCs [36]. Casticin induces apoptosis in OSCC by modulating the MAP. Additionally, mtROS can trigger the release of cytochrome C (Cyt C) by opening the mitochondrial permeability transition pore (mPTP), leading to OSCC cell apoptosis [36]. Interestingly, mitochondrial fission enhances mtROS and cytochrome C production [36]. However, certain mitochondrial characteristics also contribute to therapeutic resistance [36]. For example, mitophagy can eliminate ROS‐damaged mitochondria and reduce the efficacy of ROS‐mediated therapy in OSCC [36]. Moreover, abnormal mitochondrial nucleic acids can regulate cellular metabolism to support cancer cell survival, and factors in the TME can interact with mitochondria, further promoting resistance [36]. Thus, mitochondria play a dual role in OSCC therapy, serving as both a target and source of resistance. Targeting mitochondria through various mechanisms, including regulation of MAP and ROS levels, holds promise for improving OSCC treatment outcomes (Figure 1).

### 1.3 RNAi‐Based Therapy

Gene therapy, encompassing gene editing and silencing techniques, holds great promise for future medical treatments. One such approach is RNAi, a natural cellular mechanism for combating viral infections and regulating gene expression. RNAi therapy utilises synthetic small interfering RNAs (siRNAs) or short hairpin RNAs (shRNAs) to silence specific genes [38]. The primary objective of RNAi therapy in cancer treatment is to downregulate cell cycle and anti‐apoptotic genes in cancer cells, halting tumour proliferation and progression while sparing normal cells.

RNAi therapy has shown efficacy not only in cancer, but also in treating lung injury, respiratory diseases, cardiovascular conditions, neurodegenerative disorders and metabolic diseases [39]. In cancer treatment, RNAi therapy can be administered via plasmid‐based shRNA constructs targeting specific genes involved in tumour growth, immune response and angiogenesis. For example, RNAi therapy has been successful in inhibiting the expression of genes such as p53R2, urokinase‐type plasminogen activator receptor (u‐PAR), Bmi‐1, and l‐amino acid transporter 1 (LAT1), leading to suppression of tumour growth and metastasis [40].

The delivery of siRNAs can be achieved through various methods, including direct delivery, vector‐based delivery systems (e.g., viral vectors such as adeno‐associated viruses and lentiviruses), and nanoparticle‐based systems [41]. Although direct delivery methods are simpler, vector‐based systems offer greater stability and potency over a longer period. Nanoparticle‐based systems are specific and efficient, ensuring sustained release of siRNAs into cells [41]. However, RNAi therapy faces challenges such as off‐target effects, instability of direct delivery methods, and risk of resistance development and therapeutic failure [42]. Despite the promise of RNAi‐based therapy, several challenges hinder its clinical application, such as immune responses to viral vectors and delivery via adenoviral and lentiviral vectors can trigger an innate immune response, leading to inflammation and reduced therapeutic efficacy. To counter this, non‐viral delivery systems (e.g., lipid nanoparticles) and chemical modifications (e.g., 2′‐O‐methyl modifications) have been explored. Off‐target effects‐siRNAs may exhibit unintended gene silencing owing to partial sequence homology with non‐target transcripts. This challenge can be mitigated using bioinformatic prediction tools and chemically modified siRNAs to enhance specificity. Therapeutic resistance: Some cancer cells upregulate RNAi‐silencing pathways, thereby reducing the long‐term efficacy of RNAi therapy. Combination strategies with chemotherapeutic agents are currently being investigated to overcome this resistance. In summary, RNA interference therapy represents a promising avenue for cancer treatment and other diseases, with ongoing research aimed at optimising delivery methods and addressing the associated challenges.

### 1.4 Immunotherapy

Immunotherapy, also known as biological therapy or biological response modifier therapy, is a novel approach for treating patients with head and neck cancer. Cancer cells often evade immune surveillance by suppressing the immune system, and immunotherapy aims to counteract this by directly targeting cancer cells and boosting the immune response of the body. Various types of immunotherapy are used to treat cancer, including monoclonal antibodies targeting specific proteins such as epidermal growth factor receptor (EGFR) (e.g., cetuximab and nimotuzumab), PD‐1, and PD‐L1 (e.g., nivolumab and pembrolizumab). Cancer vaccines include protein‐based vaccines targeting proteins such as p53, mean amplitude of glycemic excursions (MAGE), and HPV, as well as cell‐based vaccines, such as dendritic cell‐based vaccines. Non‐specific immunotherapies include IL‐2, IFN‐α, IFN‐γ and IRX‐2 [43].

Systemic cell‐mediated immunotherapy, such as cytokine‐based immunotherapy, involves the administration of pro‐inflammatory cytokines to induce regional and systemic antitumor responses. Cytokines, such as GM‐CSF, IL‐2, IFN‐γ, IL‐12 and IRX‐2, have been explored for the treatment of head and neck squamous cell carcinoma (HNSCC). Key proteins, such as CTLA4 and PD‐1/PD‐L1, enable cancer cells to evade immune responses. Immunotherapy targets these proteins using anti‐CTLA4 and anti‐PD‐1/PD‐L1 agents, inhibiting T‐cell proliferation and downregulating the expression of anti‐apoptotic molecules, cytokines and the mTOR pathway in immune cells [44].

The response to immunotherapy may be slower than that to traditional chemotherapy and can sometimes lead to pseudo‐progression, where disease progression occurs before improvement. The side effects of immune checkpoint inhibitors differ from those of conventional chemotherapy and may include autoimmune reactions such as skin rashes, colitis and endocrine disturbances. Immunotherapy can be delivered locally, directly into the tumour, or systemically, targeting the whole body, potentially preventing recurrence and metastasis. However, it comes with challenges such as high cost and the need to select eligible patients and appropriate drugs based on their molecular profiles [45]. Further research is needed to explore the differences in overall survival, cancer‐free survival, recurrence rates, quality of life and adverse effects associated with various immunotherapy methods.

### 1.5 Targeting Tumour Microenvironment

Understanding the oral tumour microenvironment can provide new insights into tumour elimination. The tumour stroma and Cancer‐Associated Fibroblasts (CAFs) play pivotal roles in the development and invasion of oral malignancies. They orchestrate the recruitment of immunosuppressive cells and exhaust CD8^+^ T lymphocytes and NK cells, facilitating tumour growth and resistance to therapy. Various mechanisms within the TME, such as the recruitment of immunosuppressive cells and the expression of coinhibitory molecules, such as PD‐L1, CD155 and Tim‐3, promote cancer cell proliferation while dampening the function of anticancer immune cells. Targeting these interactions can enhance the immune response and suppress tumour growth [46]. Promising experiments have shown that depleting or reducing the number of immunosuppressive cells, such as Tumour‐Associated Macrophages (TAMs) or Myeloid‐Derived Suppressor Cells (MDSCs), can suppress oral cancer growth and enhance the effectiveness of anticancer drugs. Immunotherapy targeting co‐inhibitory molecules is a novel approach for the treatment of various tumours, including oral malignancies. Blocking molecules such as the PD‐L1/PD‐1 axis or CTLA4 can boost anticancer immunity. Combining Immune Checkpoint Inhibitors (ICIs) with other modalities, such as radiotherapy or tyrosine kinase inhibitors, has shown promise in preclinical studies, warranting further investigation in clinical trials [47]. However, there is uncertainty regarding the interactions between other cells in the oral TME. B cells, CD4+ T cells, Tregs and neutrophils have controversial effects on oral tumours, and further studies are needed to understand their roles in response to different anticancer therapies [48]. B cells play contradictory roles in the progression of OSCC. Some subsets, such as regulatory B cells (Bregs), secrete IL‐10, which contributes to an immunosuppressive microenvironment that promotes tumour growth. Conversely, activated B cells can present antigens and stimulate CD8+ T cell‐mediated cytotoxicity, thereby enhancing anti‐tumour immunity. The functional heterogeneity of B cells within the OSCC TME underscores the need for targeted strategies that selectively deplete pro‐tumorigenic subsets while preserving anti‐tumour immune responses. Considering the potential protective effects of Tregs against inflammatory responses to Human Papillomavirus (HPV), their role in HPV‐positive and HPV‐negative patients should be considered [49]. Similarly, the roles of B cells and neutrophils in promoting anticancer immunity or in developing immunosuppressive responses require careful evaluation. Targeting these cells and their interactions within the TME has the potential to improve oral cancer therapy outcomes.

### 1.6 Oral Microbiome‐Based Therapy

Oral microbiome‐based therapy is a promising avenue in the field of oral cancer treatment. Researchers are exploring innovative approaches to combat oral cancer by leveraging the intricate ecosystem of microorganisms inhabiting the oral cavity. This therapy focuses on modulating the balance and diversity of the oral microbiota to influence tumour progression and response to treatment [50]. Understanding the complex interactions between the oral microbiota and cancer cells offers the potential for developing targeted and personalised therapies. Advancements in this area hold promise for enhancing the efficacy and precision of oral cancer treatment strategies.

The oral microbiome encompasses over 600 species of bacteria, fungi, viruses, archaea and protozoa that interact within the oral cavity to form symbiotic relationships known as the oralome. While resilient, perturbations to the oral microbiome, such as those induced by tobacco and alcohol, can lead to dysbiosis, an imbalance in host–microbe interactions, potentially promoting diseases, including OSCC [51]. Previous research indicates that oral bacteria may not only contribute to oral diseases but also affect distant organs and metabolic health. Alterations in oral microbial flora may aid in the onset of cardiovascular diseases, diabetes mellitus, and respiratory diseases [52].

Studies have identified several oral pathogens enriched in OSCC tissues, such as *Porphyromonas* spp. and *Fusobacterium* spp., which are present in OSCC tissues compared with healthy tissues. Bacterial species such as *Porphyromonas gingivalis* , *Fusobacterium nucleatum* and *Prevotella intermedia* correlate with OSCC, which induces inflammatory responses, cellular proliferation, migration, and invasion and inhibits apoptosis, potentially through genomic alterations in host cells [53]. Table 4 provides a compilation of microbes that are enriched and reduced in the OSCC tumour microenvironment.

**TABLE 4:** List of microbes in OSCC.

Enriched microbiome in OSCC	Microbiome decreased in OSCC	References
*Fusobacterium, Peptostreptococcus, Parvimonas Porphyromonas gingivalis* *Fusobacterium nucleatum* subsp. *Polymorphum, Pseudomonas aeruginosa, Treponema denticola*, *Porphyromonas gingivalis* , *Fusobacteria Nucleatum*, *Tannarella Forsythia*, *Lactobacillus* spp., *Capnocytophaga gingivalis, Prevotella melaninogenica, Streptococcus mitis. Fusobacteria genera*	*Haemophilus, Granulicatella Aspergillus tamarii*, *Alternaria*, *Cladosporium*, *Halotolerans*, *Emericella*, *Malassezia restricta*, *Pichia anomala*, *Trametes Streptococcus, Capnocytiphaga, Neisseria, Haemophillus, Aggreggatibacter, Lautropia, Haemophillus, Neisseria, Leptotrichia, P. jejuni: P. melaninogenica* and *Prevotella pallens*	[52, 53, 54]

**FIGURE 2:**
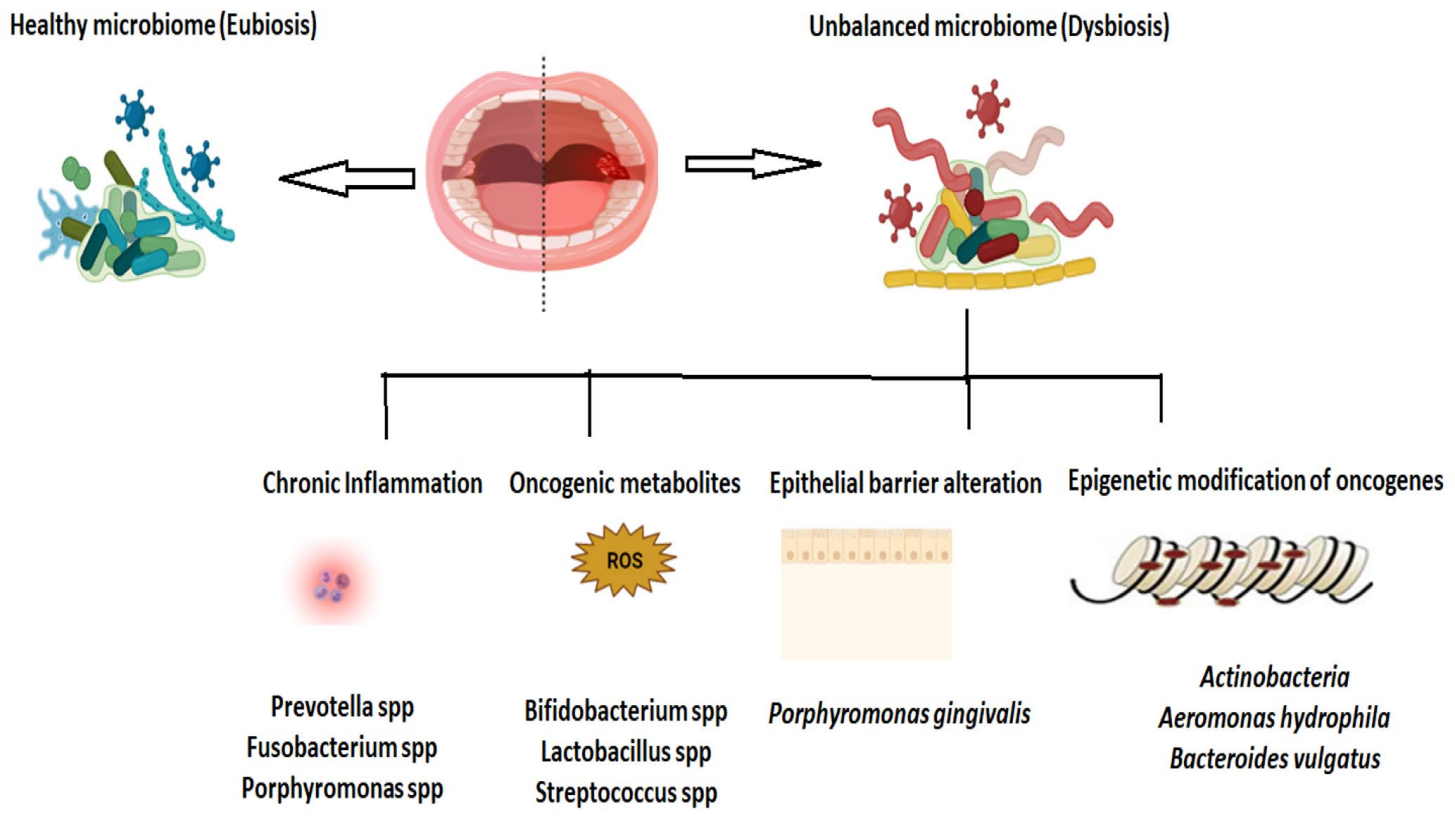
Pathogenic mechanism employed by oral microbiota during oral cancer development. Oral microbiome is involved in tumorigenesis by inducing several mechanisms such as chronic inflammation, epigenetic modification of oncogenes, etc. This figure highlights the interplay between the oral microbiome and OSCC, depicting how bacterial species such as *Porphyromonas gingivalis* and *Fusobacterium nucleatum* contribute to tumour progression through inflammation, immune evasion and epigenetic modifications.

Despite a previous focus on bacterial dysbiosis, OSCC is also linked to dysbiosis in the oral mycobiome and virome. Studies have shown that *Candida albicans* promotes tumorigenesis by inducing pro‐inflammatory T‐helper 17 cells and IL‐6 and IL‐8 cytokines [55]. Dysregulation of the oral microbiome results in chronic inflammation and epigenetic modification and promotes epithelial barrier dysfunction, which results in tumour formation. Bacterial‐induced oncogenesis involves manipulation of host cell biology, which alters tissue stem cell homeostasis. Oxidative DNA damage by bacteria‐secreted endotoxins also plays a major role in the carcinogenesis process [56] (Figure 2).

Currently, it is acknowledged that oral microorganisms play a significant role in the malignant transformation and progression of tumours, potentially serving as biomarkers for tumorigenesis. However, their role is complex, as some microorganisms may both promote and inhibit tumour growth depending on their interactions with the host and tumour microenvironment. Exploring the potential of beneficial microorganisms against cancer and understanding their intricate interactions with the host and tumour microenvironment offers promising avenues for expanding cancer treatment strategies.

### 1.7 Hormone Based Therapy

The hormonal environment plays a crucial role in tumour development, as evidenced by statistical data indicating that oral cancer is twice as common in men as it is in women. This discrepancy is often attributed to the higher rates of alcohol and tobacco use among men, which are major risk factors for the disease. Despite the significant influence of Western lifestyles on behaviour, the incidence of oral cancer among females remains low. This lower incidence is believed to be due to hormonal defence mechanisms and specific metabolic activities that differ between the sexes. The presence and expression levels of sexual hormone receptors in certain tumours underscore their potential roles in cancer pathogenesis [57].

Studies have highlighted crosstalk between oestrogen receptors (ERs) and epidermal growth factors, contributing to tumour advancement and poor prognosis. Few reports on the role of sex hormones in oral cancer have noted distinct metabolic patterns of hormones in patients with oral cancer compared to healthy individuals [58]. Other hormones, such as luteinizing hormone, follicle‐stimulating hormone and progesterone, also affect oral cancer pathogenesis and influence the overall survival of patients with head and neck cancer [58]. The assessment of ER, AR and PR expression in OSCC warrants careful consideration because of the potential variations in clinicopathological features and their correlation with different expression patterns. Oestrogen, recognised as carcinogenic, exerts its tumorigenic effects through multiple mechanisms, including the induction of chromosomal instability, leading to aneuploidy and oral cancer development. Although ER expression may represent a rare risk factor for OSCC, PR expression appears to lack relevance in OSCC development [59]. AR's ability to bind to specific DNA sequences and influence gene transcription suggests its potential involvement in the pathogenesis of salivary duct carcinoma, akin to its role in prostate carcinoma. Androgens have been shown to affect the expression of proto‐oncogenes such as c‐myc and apoptotic factors in various tissues, indicating a broader role in cancer pathogenesis and progression [60].

In patients with oral cancer, upregulated oestrogen receptor ER‐α/β and aromatase accumulation, along with downregulated progesterone receptor expression, are common. Upregulation of the nuclear oestrogen receptors ER alpha and beta, which inhibit progesterone receptor expression, correlates positively with cell proliferation and lower survival rates. The integration of gene profiling techniques with molecular approaches holds promise for enabling comprehensive studies in the future, facilitating the diagnosis and prevention of OSCC progression [61]. Although hormonal regulation in patients with oral cancer has been studied, its precise role remains unclear, necessitating further research to elucidate the interaction of hormonal receptors in oral cancer pathogenesis. Screening for various stimulating hormones may offer insights into the influence of sex hormones on oral cancer, aiding early prevention and diagnosis. Table 5 provides an overview of hormonal drugs used in cancer therapy, detailing their mechanisms of action and targeted cancer types.

**TABLE 5:** List of hormone drugs and their mode of action.

Hormone drug	Mode of action	References
Tamoxifen, toremifene	Oestrogen receptor modulator	[62]
Goserelin, leuprorelin	Luteinizing hormone analogue	[63]
Fulvestrant	Oestrogen receptor down‐regulator	[64]
Anastrozole, letrozole, Exemestane	Aromatase inhibitors	[65]

## 2 Nanoparticle‐Based Carrier for OSCC Therapy

**FIGURE 3:**
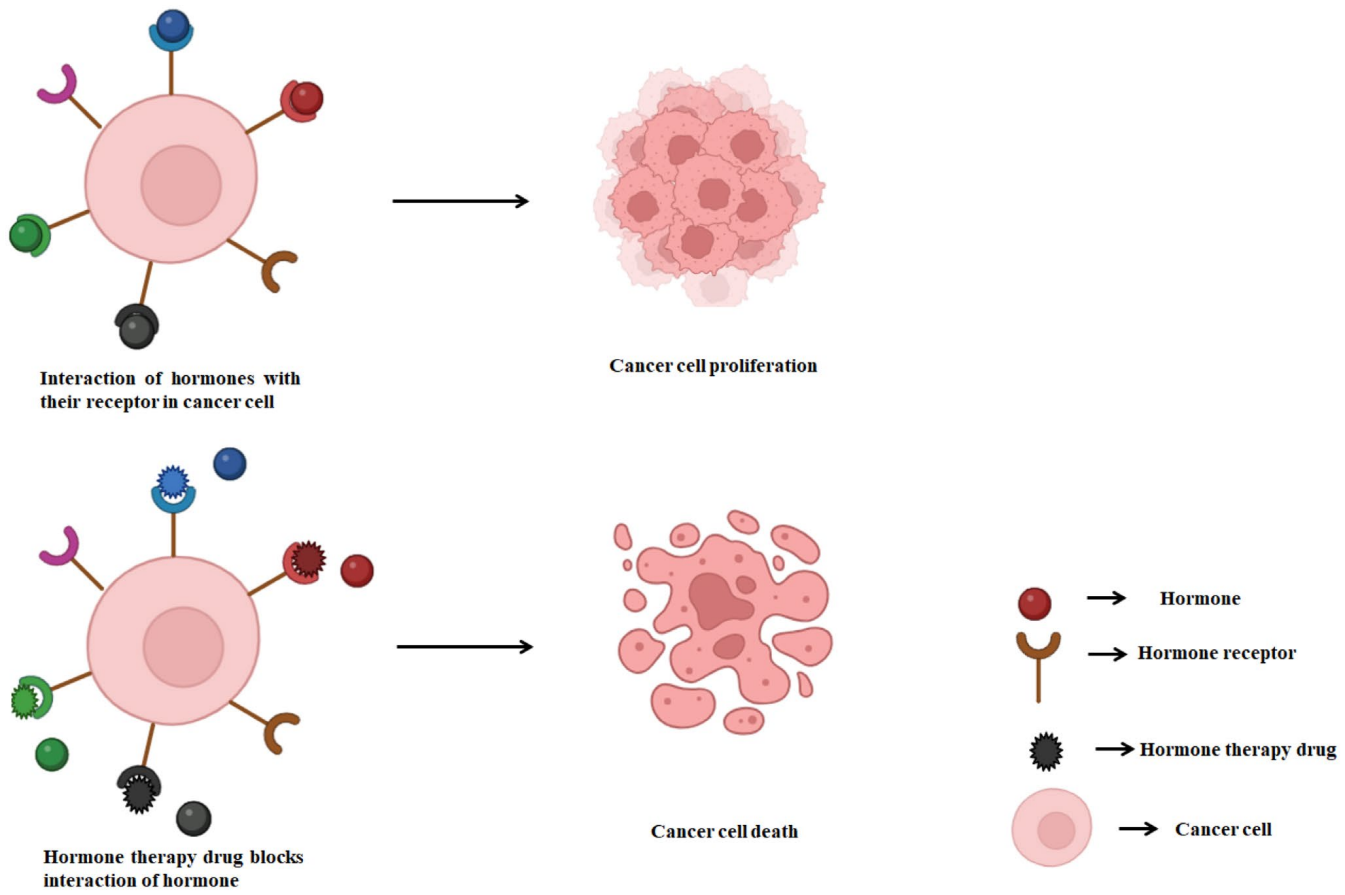
Hormone therapy for the treatment of cancer. Assessing hormone receptor and blocking the interaction results in cancer cell death. This figure provides an overview of different nanoparticle‐based drug delivery approaches, including polymeric, inorganic and combinational nanoparticles, demonstrating their advantages in improving targeted therapy, minimising side effects and overcoming drug resistance in OSCC.

To overcome the limitations of conventional chemotherapeutic drugs, there is a pressing need for molecularly targeted therapies that enhance drug efficacy and minimise toxicity. Novel controlled nanodelivery systems offer a promising solution by utilising drug‐loaded nanoparticles that can adjust drug release in response to slight changes in the microenvironment, thus enabling targeted therapy [66]. Nanotechnology‐based drug carriers, as demonstrated by studies, have facilitated selective approaches for treating OSCC [67] (Figure 3).

### 2.1 Polymeric Nanoparticles for Oral Cancer Therapy

Naturally derived synthetic polymers, such as polysaccharides, polycaprolactone (PCL), poly (lactic acid) (PLA), poly (glycolic acid) (PGA) and polyethylene glycol (PEG), serve as suitable biomaterials for preparing polymeric nanoparticles. Endo et al. developed polymeric nanoparticles to reduce cisplatin toxicity and enhance OSCC therapy using a PEG‐poly (glutamic acid) block copolymer [68]. These cisplatin‐loaded nanoparticles activated the caspase‐3 and caspase‐7 pathways, induced apoptosis, and effectively killed oral cancer cells [69]. Li et al. designed an effective chemotherapeutic system for the co‐delivery of the anticancer drug sodium arsenite (NaAsO_2_) and MTH1 inhibitor TH287 for OSCC therapy [70].

### 2.2 Inorganic Nanoparticles for Oral Cancer Therapy

Inorganic nanoparticles, characterised by low toxicity, high tolerance to organic solvents, and good bioavailability, have been extensively employed in the diagnostic and therapeutic fields for tumours with high efficacy, particularly for their unique photothermal functions in oral cancer therapy [71]. Few studies have described the design of anti‐epithelial growth factor receptor (EGFR) antibody‐conjugated gold (Au) nanoparticles for therapeutic application in OSCC therapy [72]. In vitro experiments demonstrated that OSCC cells required low energy to produce photothermal destruction for anti‐EGFR/Au conjugates, and clinical results showed effective delivery of anti‐EGFR/Au conjugates into malignant cells with deep penetration using near‐infrared (NIR) laser light [73]. Inorganic nanoparticle systems, particularly photodynamic therapy (PDT) strategies, are beneficial for oral cancer and require deep penetration of antitumor drugs in clinical practice [74].

### 2.3 Combinational (Polymeric‐Inorganic) Nanoparticles for Oral Cancer Therapy

**FIGURE 4:**
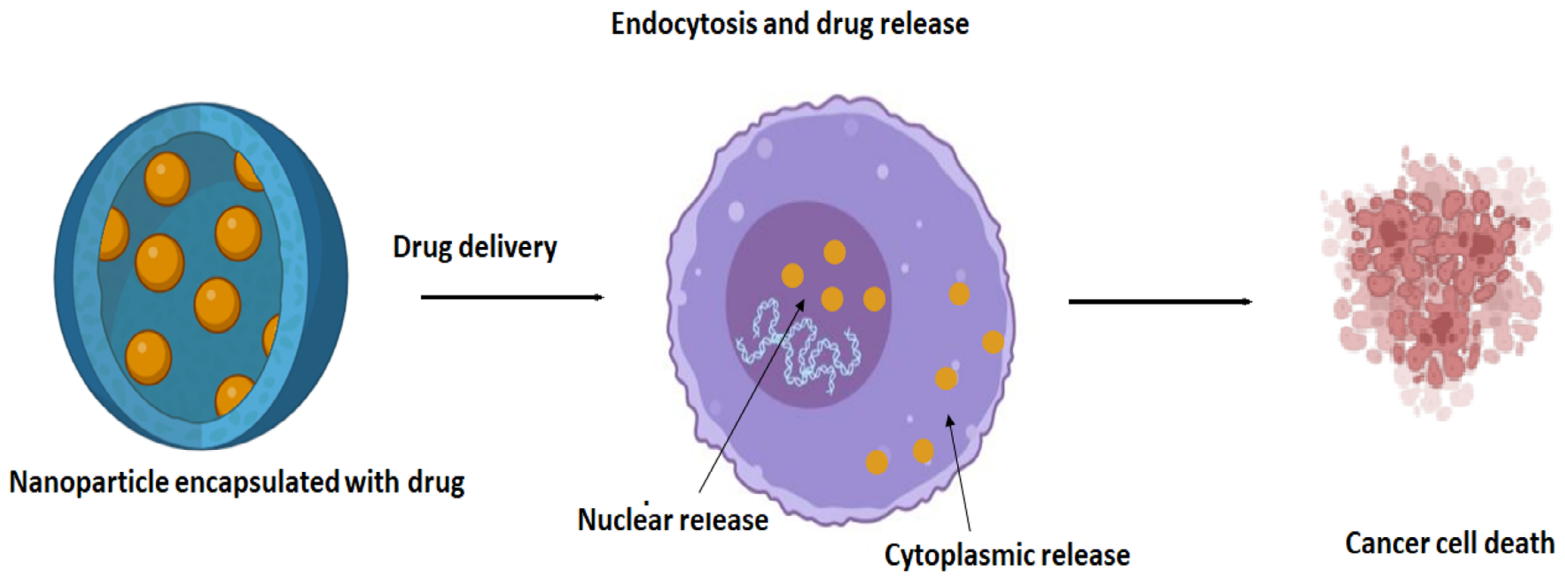
Schematic diagram for nanoparticle‐mediated drug delivery. Drugs are encapsulated in organic and inorganic nanoparticles and absorbed by the cells via endocytosis.

Combination drug treatment offers advanced therapeutic benefits through targeted drug delivery systems, reduced toxicity, and improved therapeutic efficacy. Darwish et al. developed a combinational chemo‐photothermal therapy with vincristine (VCR) as a phytochemical anticancer agent and plasmonic gold nanorods (GNRs) as photothermal reagents for OSCC therapy [75]. Recent studies have highlighted nanoparticle‐based combination therapies that integrate the photothermal effects with chemotherapy to enhance OSCC treatment [75]. Gold nanorods conjugated with vincristine (VCR) and near‐infrared (NIR) laser exposure have demonstrated synergistic tumour cell destruction via heat‐mediated drug release [76]. Similarly, polymeric nanoparticles co‐delivering sodium arsenite (NaAsO_2_) and TH287 show promise in improving the therapeutic efficacy in resistant OSCC cells [77]. These approaches leverage multimodal therapeutic strategies to overcome drug resistance and enhance targeted therapies. Table 6 provides a structured overview of the major molecular targets involved in OSCC therapy. It categorises key therapeutic targets based on their biological mechanisms and outlines the corresponding therapeutic strategies currently being explored (Figure 4).

**TABLE 6:** Key molecular targets in OSCC therapy and their mechanisms.

Target	Mechanism of action	Therapeutic strategy	References
Epigenetic modifiers	DNA methylation & histone deacetylation regulate oncogene expression	DNMT inhibitors (Decitabine), HDAC inhibitors (Vorinostat)	[78]
Mitochondrial dysfunction	Metabolic reprogramming, ROS modulation, apoptosis resistance	Mitochondrial‐targeted drugs (Metformin, Casticin)	[79]
Tumour microenvironment	Immune evasion, fibroblast activation, angiogenesis	Checkpoint inhibitors (Nivolumab, Pembrolizumab)	[80]
RNA interference (RNAi)	Post‐transcriptional gene silencing of oncogenes	siRNA‐based therapeutics	[38]
Nanoparticle drug delivery	Enhancing drug stability, specificity, and bioavailability	Gold nanoparticles, polymeric nanoparticles	[81]

## 3 Conclusion and Future Perspectives

Targeted therapies and nanotechnological advancements have significantly expanded the treatment landscape for oral cancer by offering precise drug delivery while minimising damage to healthy tissues. This review highlights various drug delivery systems, including polymeric and inorganic nanoparticles, liposomes, cyclodextrins, nanolipids, hydrogels and biomimetic formulations that address the limitations of conventional treatments. Despite their potential, clinical translation remains a challenge due to the limited number of trials, drug release control issues, and side effects. Bridging the gap between preclinical research and clinical applications is essential and requires well‐designed trials and interdisciplinary collaboration. A deeper understanding and integration of these strategies will be the key to advancing personalised and precision medicine for oral cancer management.

## Author Contributions

**Geetha Shanmugam:** methodology (equal), writing – original draft (equal). **Carmelin Durai Singh:** data curation (equal), resources (equal), software (equal). **Rekha Thiruvengadam:** data curation (equal), funding acquisition (equal), supervision (equal). **Muthu Thiruvengadam:** data curation (equal), formal analysis (equal), visualization (equal). **Vitaly Morozov:** data curation (equal), resources (equal), software (equal). **Roman Kolesnikov:** conceptualization (equal), resources (equal). **Ali Alkaladi:** project administration (equal), visualization (equal). **Rashad Saleh:** supervision (equal), writing – review and editing (equal). **Mohammad Ali Shariati:** methodology (equal), project administration (equal), resources (equal).

## Ethics Statement

As this is a review article, no new studies involving human participants or animals were conducted by the authors. All data presented were derived from previously published studies with ethical approval.

## Conflicts of Interest

The authors declare no conflicts of interest.

## Data Availability

All data supporting this review were derived from previously published studies that have been properly cited within the manuscript.
